# Near-perfect sound absorption using hybrid resonance between subwavelength Helmholtz resonators with non-uniformly partitioned cavities

**DOI:** 10.1038/s41598-024-53595-y

**Published:** 2024-02-07

**Authors:** Eunji Choi, Wonju Jeon

**Affiliations:** Department of Mechanical Engineering, Korea Advanced Institute of Science and Technology, Daejeon, 34141 Republic of Korea

**Keywords:** Mechanical engineering, Structural materials

## Abstract

We present near-perfect sound absorption using a metasurface composed of meta-atoms (MAs) which are subwavelength Helmholtz resonators (HRs) with cavities non-uniformly partitioned by membranes. By embedding the membranes at different horizontal locations in the cavities, we break geometrical symmetry between the MAs so as to derive hybrid resonance between the MAs at our target frequency. The resonance frequency of each MA is determined by delicately adjusting the locations of the membranes, resulting in perfect absorption at the target frequency which is different from the resonance frequencies of MAs. The metasurface is designed to satisfy impedance matching conditions with air at one or more target frequencies with the aid of a theoretical model for frequency-dependent effective acoustic impedance. The theoretical model is established with physical reality by considering the higher-order eigenmodes of the membrane, the visco-thermal losses in narrow orifices, and the end corrections of the subwavelength HR. The designed metasurface is fabricated and its absorption performance is verified experimentally in an impedance tube. Near-perfect absorption of sound is achieved at the target frequency of 500 Hz, which is 12.3% lower than that of near-perfect absorption by previous metasurfaces inducing hybrid resonance between HRs without membranes.

## Introduction

Wave manipulation using acoustic metamaterials and metasurfaces has been studied with great advances in acoustic clocking, impedance matching, and extraordinary absorption or insulation of sound. Perfect sound absorption is of interest to wave physicists and scientists in applied mechanics, and is important in practice for noise reduction in various mechanical systems (e.g., home appliances, mobilities, power transformers). Conventionally, porous or fibrous materials^1–3^, such as polyurethane foam, mineral wool panels, and fiberglass panels, have been used as sound absorbers for noise mitigation. Those materials dissipate the sound energy via viscous dissipation in the vicinity of the solid surface and heat conduction through the solid. They are effective in high-frequency ranges but are too bulky to absorb low-frequency sound with long-wavelengths. Other types of sound absorbers, such as Helmholtz resonators (HRs)^4–6^, micro-perforated panels (MPPs)^7–9^, and membrane resonators^10,11^, can achieve high absorption coefficients at low frequencies due to resonance but have narrow bandwidths. Several studies have combined different resonant-type absorbers^12,13^ or embedded an element such as a plate into resonators^14–17^ to obtain additionally separate absorption peaks at low frequencies. However, each peak still has narrow bandwidths or poor absorption coefficients.

Recently, several researchers have proposed subwavelength-scale acoustic metamaterials and metasurfaces for high absorption of sound at low frequencies. Acoustic metamaterials and metasurfaces composed of resonant structures, such as space-coiled structures^18–23^, decorated membrane resonators^24–27^, MPPs^28,29^ and subwavelength HRs^30–37^, have yielded high absorption coefficients at their resonance frequencies by matching the impedance of the metamaterials or metasurfaces with that of the ambient air. Among those, acoustic metasurfaces using hybrid resonance^27,35–37^ have achieved near-perfect sound absorption (over 99% absorption of sound energy). In 2014, the concept of the hybrid resonance was first demonstrated for a decorated membrane resonator composed of a cavity and an elastic membrane decorated with platelets as a phenomenon due to the strong interaction between two resonant modes of a single resonator^27^. The metasurface with a thickness of λ/133 achieved near-perfect sound absorption at 152 Hz, and was much thinner than the incident wavelength but had a narrow bandwidth of less than 5 Hz.

Hybrid resonance between adjoint resonators was also demonstrated by Li et al.^35^. They proposed a metasurface with a thickness of λ/20 which achieved near-perfect sound absorption with the aid of the hybrid resonance between two subwavelength HRs with different resonance frequencies. By considering the frequency-dependent visco-thermal effects on the narrow orifice and cavity, Ryoo and Jeon proposed a systematic design procedure for a metasurface inducing hybrid resonance between subwavelength HRs at a target frequency^36^. Here, the hybrid resonance was induced by adjusting the radii of the neck to break symmetry between the HRs. The visco-thermal effects in subwavelength HRs have the advantage of widening the bandwidth for high absorption of sound than that of the metasurface using the decorated membrane resonator. Furthermore, they proposed a metasurface based on the concept of space coiling and hybrid resonance between subwavelength HRs to improve low-frequency sound absorption^37^.

In this paper, we propose a sound-absorbing metasurface composed of meta-atoms (MAs) which are subwavelength HRs with membranes for perfect absorption of low-frequency sound with the aid of hybrid resonance. By embedding the membranes at different horizontal locations in the cavities, we break geometrical symmetry between the MAs. While, in previous studies, hybrid resonance between HRs was induced by adjusting the geometrical dimensions of HRs such as radii of the neck, in this study, hybrid resonance between the MAs is derived at a target frequency by adjusting the locations of the membranes to determine precisely the resonance frequency of each MA. A theoretical model is established to derive frequency-dependent effective acoustic impedance of the HR with the membrane. Using the theoretical model, the proposed metasurface is designed to match the acoustic impedance of the metasurface with the impedance of air at a target frequency. For physical reality, higher-order eigenmodes of the membrane and the damping effect in the membrane due to material property are taken into account. Also, the visco-thermal losses in the narrow orifices are considered by using effective materials. We analyze the absorbing mechanism of the designed metasurface via the hybrid resonance at the target frequency which is different from the resonance frequencies of the MAs. Also, we demonstrate that the designed metasurface near-perfectly absorbs the sound energy at the target frequency through experimental validation.

## Results

### Geometry of the metasurface

**Figure 1:**
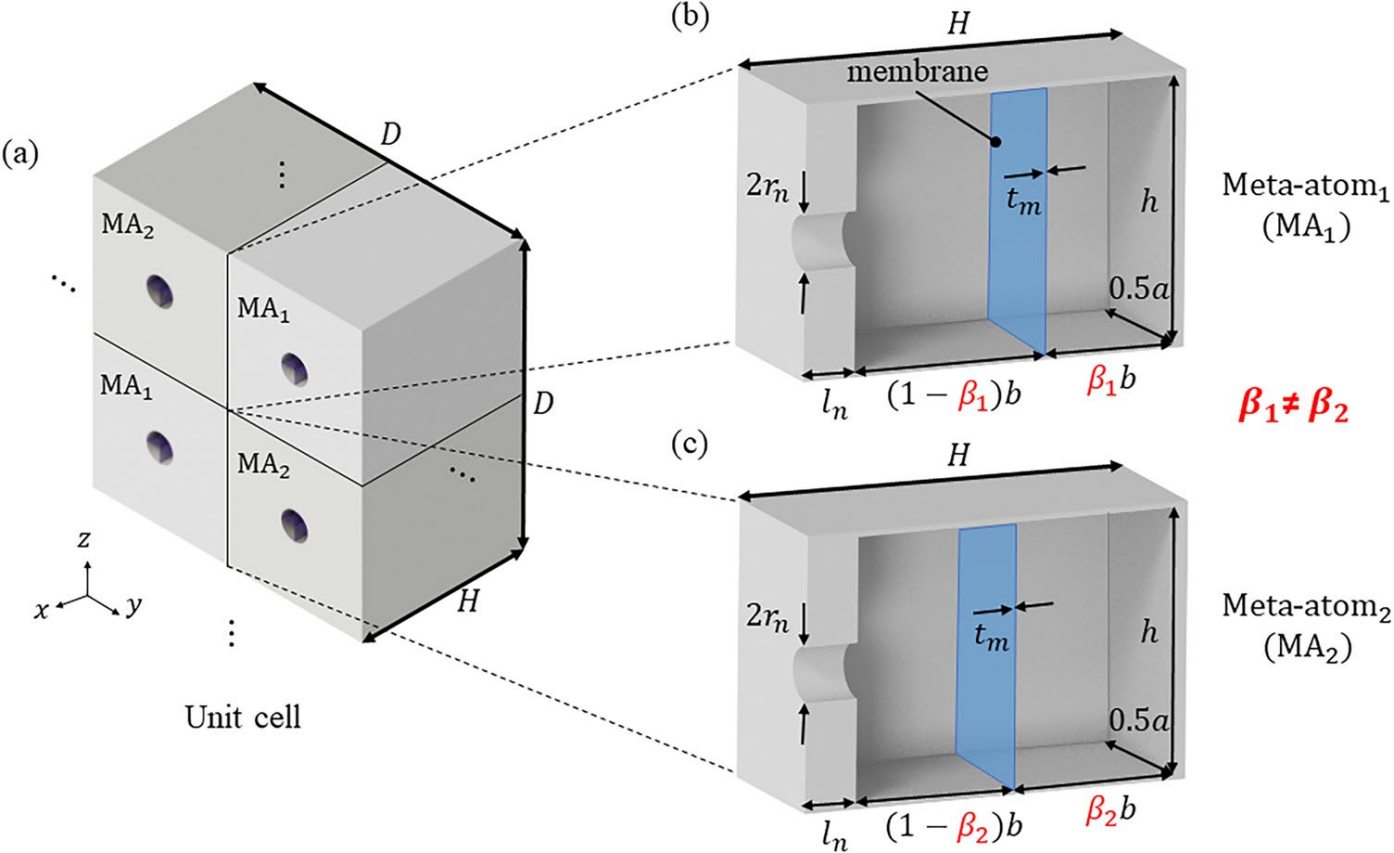
(**a**) Geometry and dimensions of a unit cell in the metasurface. (**b,c**) Vertical cross-sections of the MAs with membranes in different locations (${\beta }_{1}\ne {\beta }_{2}$).

We describe the geometry and dimensions of a sound-absorbing metasurface with a subwavelength scale, aiming to perfectly absorb low-frequency sound. The metasurface is a two-dimensional periodic array of unit cells composed of subwavelength HRs. Figure 1a illustrates the geometry and dimensions of the unit cell in the metasurface. Each HR is referred to as a meta-atom (MA) and there are two types, denoted by ${{\text{MA}}}_{1}$ and ${{\text{MA}}}_{2}$, which have different resonance frequencies. Figure 1b,c show vertical cross-sections of the two MAs. Each MA is a subwavelength HR, whose main cavity is partitioned into two cavities by a membrane embedded parallel to the bottom of the HR. In the MAs, the cavity with the aperture is called the open cavity, and the other one is called the closed cavity. ${\beta }_{j}$ for $\mathrm{each }\,{{\text{MA}}}_{j} (j = 1, 2)$ is defined as the ratio of the depth of the closed cavity to that of the entire cavity.

In previous research^36^, by adjusting the radii of the neck, hybrid resonance was induced and perfect sound absorption was achieved. That is, ${r}_{n}$ of the HRs were different. In this paper, to induce the strong interaction between the MAs due to phase difference, the cavities of the MAs are partitioned non-uniformly (i.e., ${\beta }_{1}\ne {\beta }_{2}$) while the geometrical parameters of the MAs are the same each other. Each ${\beta }_{j}$ is determined so that the hybrid resonance occurs by maximizing the interaction between the MAs with the aid of a theoretical derivation of the frequency-dependent effective acoustic impedance of the metasurface.

### Theoretical model for sound-absorbing metasurfaces

**Figure 2:**
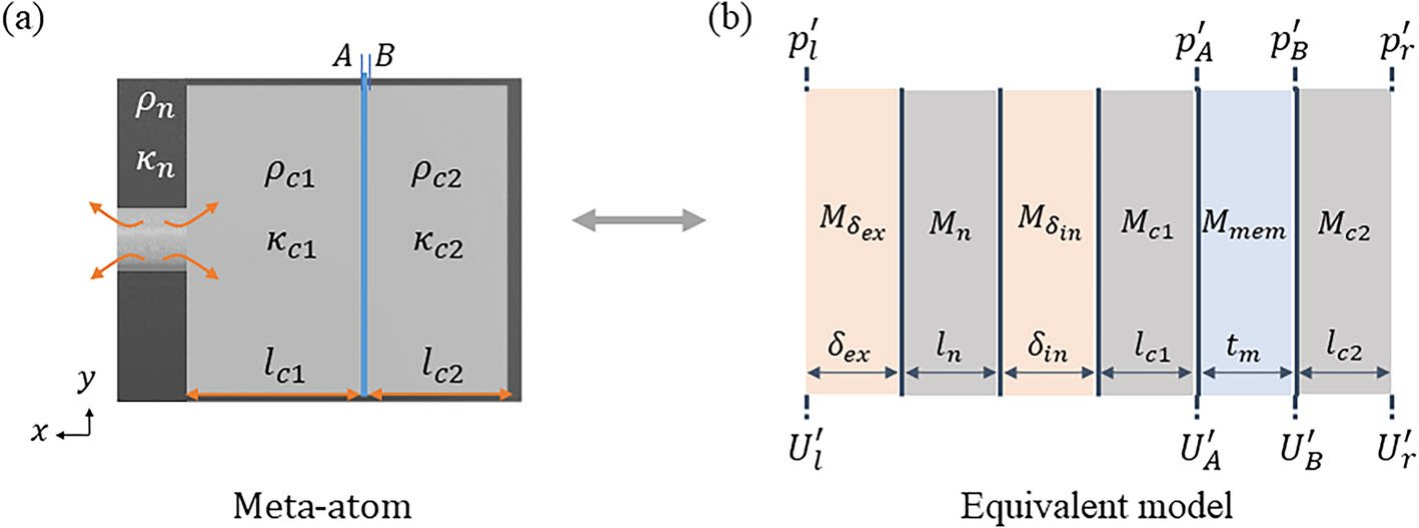
(**a**) Vertical cross-section of the MA. (**b**) Equivalent six-layer model for the MA with end corrections.

A theoretical model is established to derive the frequency-dependent effective acoustic impedance of the metasurface using the transfer matrix method. As shown in Fig. 2, each MA can be recast into equivalent six layers: the neck, both end corrections of the neck, the open cavity, the membrane, and the closed cavity, and each layer can be expressed as a transfer matrix. First, the transfer matrices ${M}_{q}$ ($q=n,c1,c2$) for the neck and both cavities are derived using the effective material properties, which consider the effects of visco-thermal losses in the subwavelength HRs. Second, the transfer matrices ${M}_{{\delta }_{p}}$ ($p=ex,in$) for the external and internal end corrections due to radiation impedances at the ends of the neck are obtained. Third, the transfer matrix ${M}_{mem}$ for the membrane is derived using its area-averaged velocity. Since the low-frequency range affected by a fundamental mode below 1 kHz is of great interest in this paper, the area-averaged velocity of the membrane is used. The area-averaged velocity of the membrane implies that it assumes that the membrane acts as a piston. The local variation in the $y$- and $z$-directions is not considered, as this is not a full three-dimensional treatment. So, although the movements of higher-order eigenmodes with out-of-phase responses are also treated like the movement of a piston with a single body, mode superposition theory is used to consider the effect of higher-order eigenmodes of the membrane on the low-frequency range. Finally, the overall transfer matrix of the MA is obtained by combining the transfer matrices obtained above.

The transfer matrices for the neck and both cavities can be written as

1 $${M}_{q}=\left(\begin{array}{cc}{\text{cos}}\left({k}_{q}{l}_{q}\right)& i{Z}_{q}{\text{sin}}({k}_{q}{l}_{q})\\ \frac{i}{{Z}_{q}}{\text{sin}}({k}_{q}{l}_{q})& {\text{cos}}\left({k}_{q}{l}_{q}\right)\end{array}\right)\, {\text{for}}\, q=n,c1,c2,$$

where ${Z}_{n, c1, c2}$ and ${k}_{n,c1,c2}$ are the effective acoustic impedances and wavenumbers of the neck, open cavity, and closed cavity, respectively. For a normally incident wave, they can be expressed as follows: ${Z}_{q}=\sqrt{{\kappa }_{q}{\rho }_{q}}/{A}_{q}$ and ${k}_{q}=\omega \sqrt{{\rho }_{q}/{\kappa }_{q}}$. Here, ${A}_{q}$ is the surface area of the neck or cavity of the MA, and ${l}_{c1}$ and ${l}_{c2}$ are defined as $\left(1-{\beta }_{j}\right)b-{t}_{m}/2$ and ${\beta }_{j}b-{t}_{m}/2$, respectively. To incorporate the effects of visco-thermal losses in the narrow neck and cavities, we use the effective material properties (mass density ${\rho }_{q}$ and bulk modulus ${\kappa }_{q}$) derived from Stinson’s model^38^.

The transfer matrices for the end corrections can be written as

2 $${M}_{{\delta }_{p}}=\left(\begin{array}{cc}1& i{\delta }_{p}{k}_{n}{Z}_{n}\\ 0& 1\end{array}\right)\, \mathrm{for }\,p=ex,in,$$

where ${\delta }_{ex, in}$ are the external and internal end corrections due to the radiation impedance at each end of the neck of the MA. The external and internal end corrections are calculated using the formulas given in previous studies^39,40^.

The area-averaged velocity of the membrane is calculated, and the transfer matrix for the membrane is thus derived. Because the membrane is too thin compared with the wavelength of the incident wave, the variation in the velocity along the *x*-direction can be ignored. It can, thus, be assumed that the velocities of both sides of the membrane are the same. Since the edges of the membrane are fixed, the tension per unit length ${T}_{j}$ on the membrane in ${{\text{MA}}}_{j}$ for $j$ = 1, 2 is assumed to remain a constant as the membrane vibrates. Then, the governing equation of the membrane excited by acoustic pressure can be expressed as

3 $$-{T}_{j}^{*}{\nabla }^{2}w\left(y,z,t\right)+{\rho }_{m}{t}_{m}\frac{{\partial }^{2}w\left(y,z,t\right)}{\partial {t}^{2}}= {p}_{A}^{\prime}{e}^{i\omega t}-{p}_{B}^{\prime}{e}^{i\omega t},$$

where ${\rho }_{m}$ is the mass density of the membrane, ${t}_{m}$ is the thickness of the membrane with 0.28 mm, and $w\left(y, z, t\right)$ is the transverse displacement of a point ($y$,$z$) on it in the $x$-direction at time *t*. ${p}_{A}^{\prime}{e}^{i\omega t}$ and ${p}_{B}^{\prime}{e}^{i\omega t}$ are the acoustic pressures on the upper and lower sides of the membrane, respectively. ${T}_{j}^{*}$[$={T}_{j}\left(1+i{\eta }_{m}\right)]$ is the complex tension of the membrane where ${\eta }_{m}$ is loss factor. Using mode superposition theory to consider the higher-order eigenmodes as well as the fundamental eigenmode, the displacement $w\left(y,z,t\right)$ of the membrane can be written as

4 $$w\left(y,z,t\right)={w}^{*}\left(y,z\right){e}^{i\omega t}=\sum \limits_{m,n}{W}_{mn}\left(y,z\right){q}_{mn}{e}^{i\omega t},$$

where ${q}_{mn}$ is an unknown constant to be determined below, ${w}^{*}\left(y,z\right)$ is the amplitude of the displacement, and ${W}_{mn}(y,z)$ is the mode shape function with fixed edges as follows:

5 $${W}_{mn}\left(y,z\right)={\text{sin}}\left(\frac{m\pi }{a}y\right)\cdot {\text{sin}}\left(\frac{n\pi }{h}z\right)\,\mathrm{ for }\,m=1, 2, \dots {\text{and}} \,n=1, 2,\dots ..$$

By substituting Eq. (4) into Eq. (3), multiplying both sides of the equation by the mode shape function ${W}_{kl}(y,z)$, and integrating both sides over the area of the membrane, Eq. (3) can be reduced due to the orthogonality of the mode shape functions as follows:

6 $$-{\upomega }^{2}{M}_{mn}{q}_{mn}+{K}_{mn}{q}_{mn}=\left({p}_{A}^{\prime}-{p}_{B}^{\prime}\right){B}_{mn}\,\mathrm{ for }\,m=1, 2, \dots , 5 \,{\text{and}}\, n=1, 2,\dots , 5,$$

where

7 $${M}_{mn}={\rho }_{m}{t}_{m}\int \limits_{S}{W}_{mn}^{2}dS,$$

8 $${K}_{mn}=-{T}_{j}^{*}\int \limits_{S}{W}_{mn}{\nabla }^{2}\left({W}_{mn}\right)dS,$$

9 $${B}_{mn}=\int\limits_{S}{W}_{mn}dS.$$

${M}_{mn}$, ${K}_{mn}$, and ${B}_{mn}$ are the equivalent mass, stiffness, and pressure weight of the membrane for mode (*m, n*). The unknown constant ${q}_{mn}$ can be determined by solving Eq. (6) above. In this paper, only modes up to (5,5) are considered because the frequencies of subsequent modes (5,5) are very high compared to 1 kHz and do not affect the sound absorption coefficients below 1 kHz. The amplitude of the area-averaged velocity ${\overline{v} }_{mem}\left(y,z\right)$ of the membrane can then be calculated as:

10 $${\overline{v} }_{mem}\left(y,z\right)=\frac{i\omega \int\limits_{S}{w}^{*}\left(y,z\right)dS}{{A}_{c1}}=\frac{\left({p}_{A}^{\prime}-{p}_{B}^{\prime}\right)i\omega \varepsilon }{{A}_{c1}},$$

where

11 $$\varepsilon =\sum\limits_{m,n}\left(\frac{{B}_{mn}^{2}}{{K}_{mn}-{\upomega }^{2}{M}_{mn}}\right).$$

According to the assumption that the velocities on both sides of the membrane are the same (${U}_{A}^{\prime}={U}_{B}^{\prime}{=\overline{v} }_{mem}\left(y,z\right)$, where ${U}_{A,B}^{\prime}$ are the acoustic velocities on the upper and lower sides of the membrane), the transfer matrix ${M}_{mem}$ for the membrane layer can be obtained as follows:

12 $${M}_{mem}=\left(\begin{array}{cc}1& 1/i\omega \varepsilon \\ 0& 1\end{array}\right).$$

The transfer matrix for each MA can be written as

13 $$\mathbf{T}=\left(\begin{array}{cc}{T}_{11}& {T}_{12}\\ {T}_{21}& {T}_{22}\end{array}\right)=\boldsymbol{ }{M}_{{\delta }_{ex}}{M}_{n}{M}_{{\delta }_{in}}{M}_{c1}{M}_{mem}{M}_{c2}.$$

Then,

14 $$\left(\begin{array}{c}{p}_{l}^{\prime}\\ {A}_{l}{U}_{l}^{\prime}\end{array}\right)=\mathbf{T}\left(\begin{array}{c}{p}_{r}^{\prime}\\ {A}_{r}{U}_{r}^{\prime}\end{array}\right),$$

where ${p}_{l,r}^{\prime}$ and ${U}_{l,r}^{\prime}$ are the acoustic pressures and velocities on the metasurface and the bottom of the MA, respectively, and ${A}_{l}$ and ${A}_{r}$ are the cross-sectional areas of the unit cell and the cavity, respectively. The bottom surface of the MA has an acoustically rigid boundary (${U}_{r}^{\prime}=0).$ Thus, the effective acoustic impedance of each MA is calculated as ${Z}_{MA}={T}_{11}/{T}_{21}$ from Eq. (14) using the transfer matrix method. The effective acoustic impedance ${Z}_{MS}$ of the unit cell composed of multiple MAs is

15 $${Z}_{MS}={\left(\sum\limits_{j=1}^{N}{Z}_{MA,j}^{-1}\right)}^{-1},$$

where ${Z}_{MA,j}$ is the effective acoustic impedance of the $j{\text{th}}$ MA. As indicated in Eq. (15), ${Z}_{MS}$ is determined by a combination of MAs regardless of their arrangement because the metasurface consists of unit cells with subwavelength scale. The sound absorption coefficient ${\alpha }_{MS}$ of the metasurface can now be calculated:

16 $${\alpha }_{MS}\left(\theta \right)=1-{\left|\frac{{Z}_{MS}{\text{cos}}\theta -{Z}_{0}}{{Z}_{MS}{\text{cos}}\theta +{Z}_{0}}\right|}^{2}=1-{R}^{2},$$

where ${Z}_{0}(=\sqrt{{\kappa }_{0}{\rho }_{0}}/{D}^{2})$ is the effective acoustic impedance of air, and *R* is the reflection coefficient of the metasurface. $\theta$ is the angle of incidence of sound, which is assumed to be normally incident, i.e., $\theta =0.$ To achieve perfect sound absorption (i.e., ${\alpha }_{MS}=1)$, we design the metasurface so that the reflectance ${R}^{2}$ is zero.

### Design of a metasurface for perfect absorption

We design a metasurface by using the theoretical model established in the previous section. When we match the impedance of the metasurface to that of the ambient air, the reflectance becomes zero, and those impedance matching conditions are expressed by the following two equations:

17 $${\text{Re}}\left[{Z}_{MS}\right]/{Z}_{0}-1=0,$$

18 $${\text{Im}}\left[{Z}_{MS}\right]/{Z}_{0}=0,$$

where ${\text{Re}}\left[{Z}_{MS}\right]$ is the real part of ${Z}_{MS}$, which is the effective acoustic resistance of the unit cell, and ${\text{Im}}\left[{Z}_{MS}\right]$ is its imaginary part, which is the effective acoustic reactance of the unit cell. To satisfy Eqs. (17) and (18), an objective function ${F}_{obj}$ has to be minimized at the given target frequency ${f}_{target}$:

19 $${\mathrm{min }F}_{obj}\left(a,h,b,{l}_{n},{r}_{n},D,H, {f}_{target},{T}_{1},{T}_{2},{\beta }_{1},{\beta }_{2}\right)={\left\{{\text{Re}}\left[{Z}_{MS}\left({f}_{target}\right)\right]/{Z}_{0}-1\right\}}^{2}+{\left\{{\text{Im}}\left[{Z}_{MS}\left({f}_{target}\right)\right]/{Z}_{0}\right\}}^{2},$$

where $a$, $h$, $b$, ${l}_{n}$, ${r}_{n}$, $D$, and $H$ are the given geometrical parameters. ${T}_{j}$ and ${\beta }_{j}$ for $j$ = 1, 2 are the tension and location of the membrane in each MA, respectively.

The fixed geometrical variables of the metasurface were determined as follows: $a$ = $h$ = 25 mm, $b$ = 35 mm, ${l}_{n}$ = 4 mm, ${r}_{n}$ = 2.5 mm, $D$ = 88.6 mm, and $H$ = 40 mm. Both tensions were fixed at 45 N/m, which was determined so that the target frequency is within the range of the resonance frequency of the MA according to the locations of the membranes, as the tension governs the resonance frequency. An optimization method (the sequential quadratic method) is used to minimize the objective function obtained from the theoretical model at the target frequency of 500 Hz and satisfy the impedance matching conditions. Then, the values of ${\beta }_{j}$ for $j$ = 1, 2 were obtained from the optimization method as follows: (${\beta }_{1}, {\beta }_{2}$) = (0.49, 0.60), which were rounded to two decimal places.

### Near-perfect sound absorption of the metasurface using hybrid resonance

We describe the theoretical results and explain how the metasurface can achieve near-perfect sound absorption using hybrid resonance at the target frequency. Firstly, we obtain the sound absorption spectrum from the theoretical model and present the design results. Next, we analyze the absorbing mechanism via hybrid resonance by performing FE simulation.

**Figure 3:**
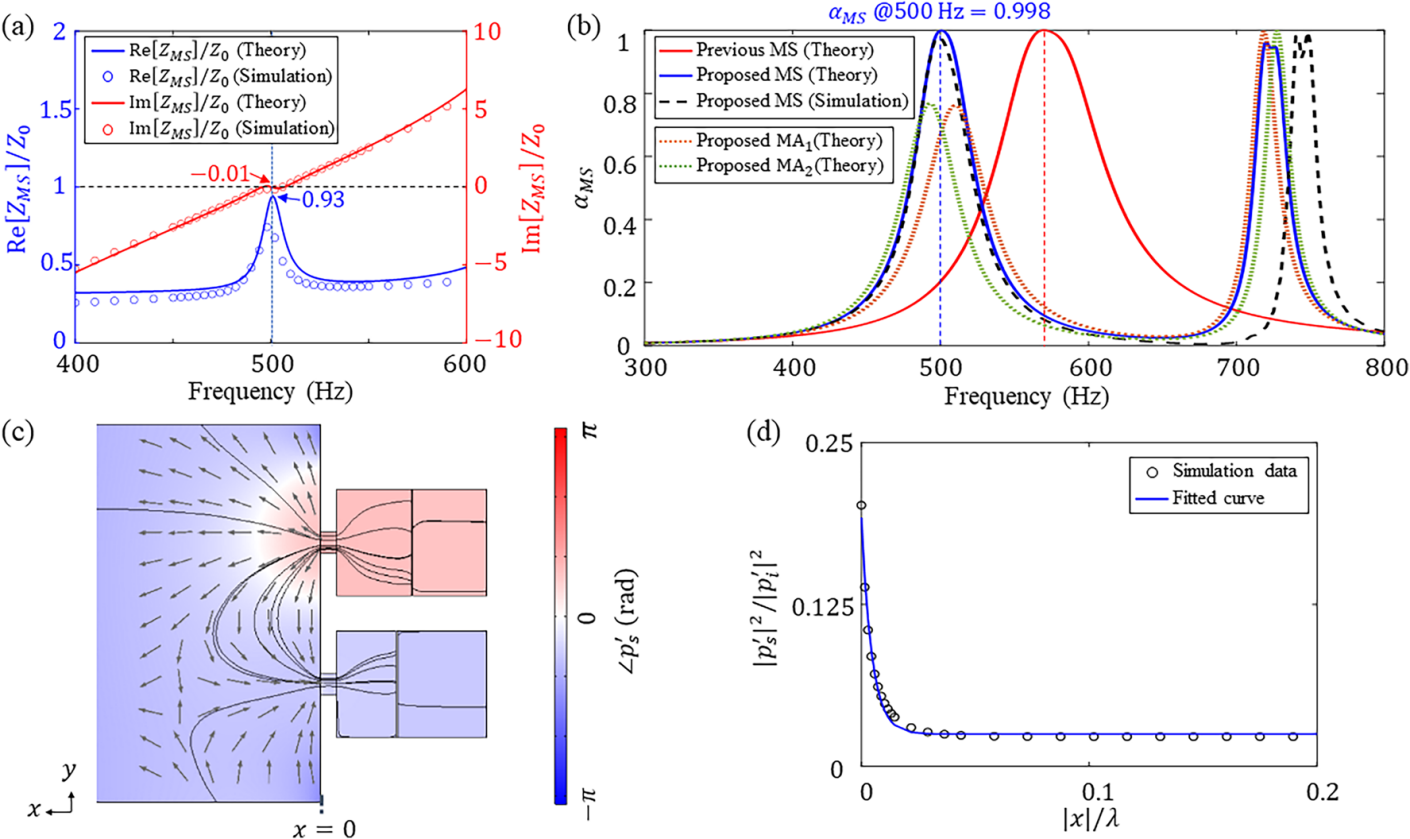
(**a**) Normalized effective acoustic resistances and reactances and (**b**) the sound absorption spectra of the designed metasurface (MS) with (${\beta }_{1}$, ${\beta }_{2}$) = (0.49, 0.60) obtained using the theoretical model and the FE simulation. The normalized impedance of the metasurface at 500 Hz (${\text{Re}}\left[{Z}_{MS}\right]/{Z}_{0}$, ${\text{Im}}\left[{Z}_{MS}\right]/{Z}_{0}$) is (0.93, − 0.01) and the corresponding absorption coefficient is 0.998. For comparison, the sound absorption spectrum of the previous metasurface using the HRs without the membranes is also displayed together. The orange and green dotted lines indicate the sound absorption spectra of the proposed ${{\text{MA}}}_{1}$ and ${{\text{MA}}}_{2}$, respectively. (**c**) Phase contour plot of the scattered acoustic pressure (${p}_{s}^{\prime}$) with the streamlines for the scattered acoustic velocity (${{\varvec{u}}}_{{\varvec{s}}}^{\boldsymbol{{\prime}}}$). The gray arrows indicate directions of the scattered acoustic velocities. (**d**) Scattered sound energy normalized by the incident sound energy versus distance |*x*| from the acoustic metasurface normalized by the wavelength ($\lambda$) of the incident sound.

The sound absorption coefficients of the designed metasurface were calculated using the theoretical model and the FE simulation. Figure 3a,b show the normalized effective acoustic resistance (blue line) and reactance (red line) of the designed metasurface and the sound absorption spectra of the designed metasurface, respectively. As shown in Fig. 3a, the impedance matching conditions were almost satisfied at the target frequency (${\text{Re}}[{Z}_{MS}]/{Z}_{0}=0.93$ and ${\text{Im}}[{Z}_{MS}]/{Z}_{0}=-0.01$), resulting in an absorption coefficient of 0.998 at 500 Hz. For the result of ${\text{Re}}\left[{Z}_{MS}\right]/{Z}_{0} <1$ at the target frequency of 500 Hz, we confirmed that it was possible to satisfy the impedance matching condition at 500 Hz when considering the optimal values to three decimal places. We used the rounded values for two reasons; one was the ease for the fabrication (with two decimal places instead of three) and the other was the achievement of sufficiently high absorption (greater than 0.99). The absorption band of the designed metasurface for half maximum coefficient was between 480 and 525 Hz. The related indicator, $Q$-factor, is defined as the ratio of the first peak frequency to the full width at half maximum (i.e., ${f}_{1st\;peak}/\Delta {f}_{@\alpha =0.5\cdot {\alpha }_{1st\;peak}}$ where ${\alpha }_{1st\;peak}$ is the sound absorption coefficient at the first peak frequency). In the case of the designed metasurface, ${f}_{1\mathrm{st\;peak}}$ was 500 Hz and $\Delta {f}_{@\alpha =0.5\cdot {\alpha }_{1st\;peak}}$ was $\Delta {f}_{@\alpha =0.50}=45$ Hz, and the corresponding $Q$-factor of the designed metasurface was 11.1. Figure 3b also compares the sound absorption spectra of the proposed metasurface and a previous metasurface^36^ which induced hybrid resonance between subwavelength HRs without membranes by adjusting the radii of the neck. In Fig. 3b, the vertical dashed lines indicate the first absorption peaks in sound absorption spectra of the previous and proposed metasurfaces. For a fair comparison, the size of the cavity and the length and total cross-sectional area of the neck in the previous metasurface were constrained to be the same as those of the proposed metasurface. As a result of optimization within those conditions, near-perfect absorption of sound was achieved at 570 Hz with a bandwidth of 83 Hz using the previous metasurface. The corresponding $Q$-factor for the first peak frequency was 6.9. As shown in Fig. 3b, the $Q$-factor of the proposed metasurface was higher than the $Q$-factor of the previous metasurface because a membrane with a very high $Q$-factor was added. Nevertheless, the purpose of membranes is to realize low-frequency sound absorption since they are effective in lowering the resonance frequency of a HR. Due to the lower effective stiffness of the MA in the proposed metasurface compared to that of the previous one, it was possible to absorb more than 99% of incident sound energy at a target frequency which is 12.3% (70 Hz) lower than that of the previous metasurface without membranes. In other words, the proposed metasurface can absorb sound energy with a more compact size compared to the previous one in given space.

For the metasurface to absorb sound energy perfectly, the impedance matching conditions must be satisfied, and the incident energy needs to be dissipated through a proper mechanism. In this paper, we utilize the unique characteristics of sound waves in narrow orifices of the subwavelength HRs that constitutes the metasurface for sound absorption. First, the acoustic and thermal boundary layers developed in the narrow orifices facilitated the sound energy dissipation due to visco-thermal losses in the neck because the thicknesses of those boundary layers are not negligible but comparable to the dimension of neck diameters. Second, those visco-thermal losses in the neck induced a strong interaction between the meta-atoms in the way of deriving horizonal streamlines, which resulted in out-of-phase reflection leading to destructive interference between waves emanated from the necks. In Fig. 3b, the orange and green dotted lines indicate the sound absorption spectra of the proposed ${{\text{MA}}}_{1}$ and ${{\text{MA}}}_{2}$, respectively. The absorption peak of the metasurface occurs between the resonance frequencies of the MAs, which indirectly shows that the phase difference of MAs induced the hybrid resonance. Figure 3c shows a phase contour plot of the scattered acoustic pressure (${p}_{s}^{\prime}$) with streamlines for the scattered acoustic velocity (${{\varvec{u}}}_{{\varvec{s}}}^{\boldsymbol{{\prime}}}$) calculated from the FE simulation in a vertical cut-plane at 500 Hz. In Fig. 3c, the gray arrows indicate the directions of the scattered acoustic velocities. As shown in Fig. 3c, a strong interaction between the meta-atoms in the way of deriving horizonal streamlines results in out-of-phase reflection. This leads to destructive interference between waves emanated from the necks. Figure 3d shows the scattered sound energy normalized by the incident sound energy versus distance |*x*| from the acoustic metasurface normalized by the wavelength ($\lambda$) of incident sound at 500 Hz. The scattered sound energy was calculated along the positive *x*-axis as indicated in Fig. 3c*.* The reflected wave energy exponentially decays along the *x*-direction, as plotted in Fig. 3d, implying that the phase difference causes destructive interference between the waves reflected from each MA in the far field. As a result of those two phenomena (sound energy dissipation due to the visco-thermal losses in the neck and destructive interference between waves reflected from MAs), the metasurface has the higher absorption coefficients and wider bandwidth than those of each MA, indicating that near-perfect absorption of sound can be achieved using the proposed metasurface with the low $Q$-factor compared to a single resonator.

### Experimental validation

**Figure 4:**
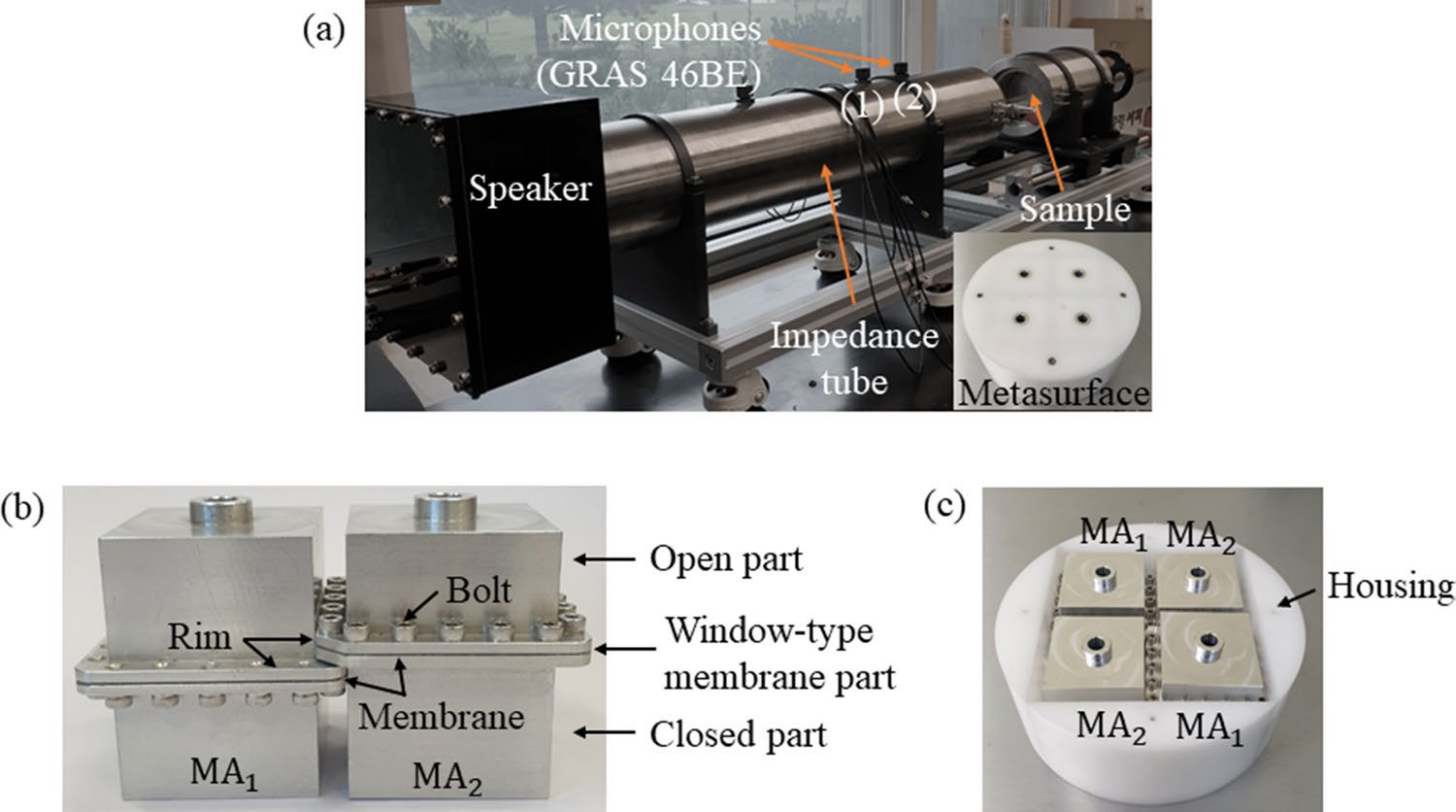
Photographs of (**a**) the experimental setup with the circular impedance tube, (**b**) the separately fabricated MAs with the membranes bolted in place, and (**c**) the fabricated unit cell assembled with the MAs with the cover removed.

The experiments to measure the sound absorption coefficients are described in this section, including the fabrication of the metasurface and the experimental setup. Figure 4a is a photograph of the experimental setup for measuring the sound absorption coefficient using a circular impedance tube. The MAs shown in Fig. 4b were assembled from three parts: an open part including the neck and the open cavity, a closed part including the closed cavity, and a window-type membrane part consisting of the rim and the membrane. The membranes were bolted between two rims of the window-type membrane parts to ensure that the boundary conditions are fixed. The unit cell was then assembled from the separately fabricated MAs and a circular housing, as shown in Fig. 4c.

**Figure 5:**
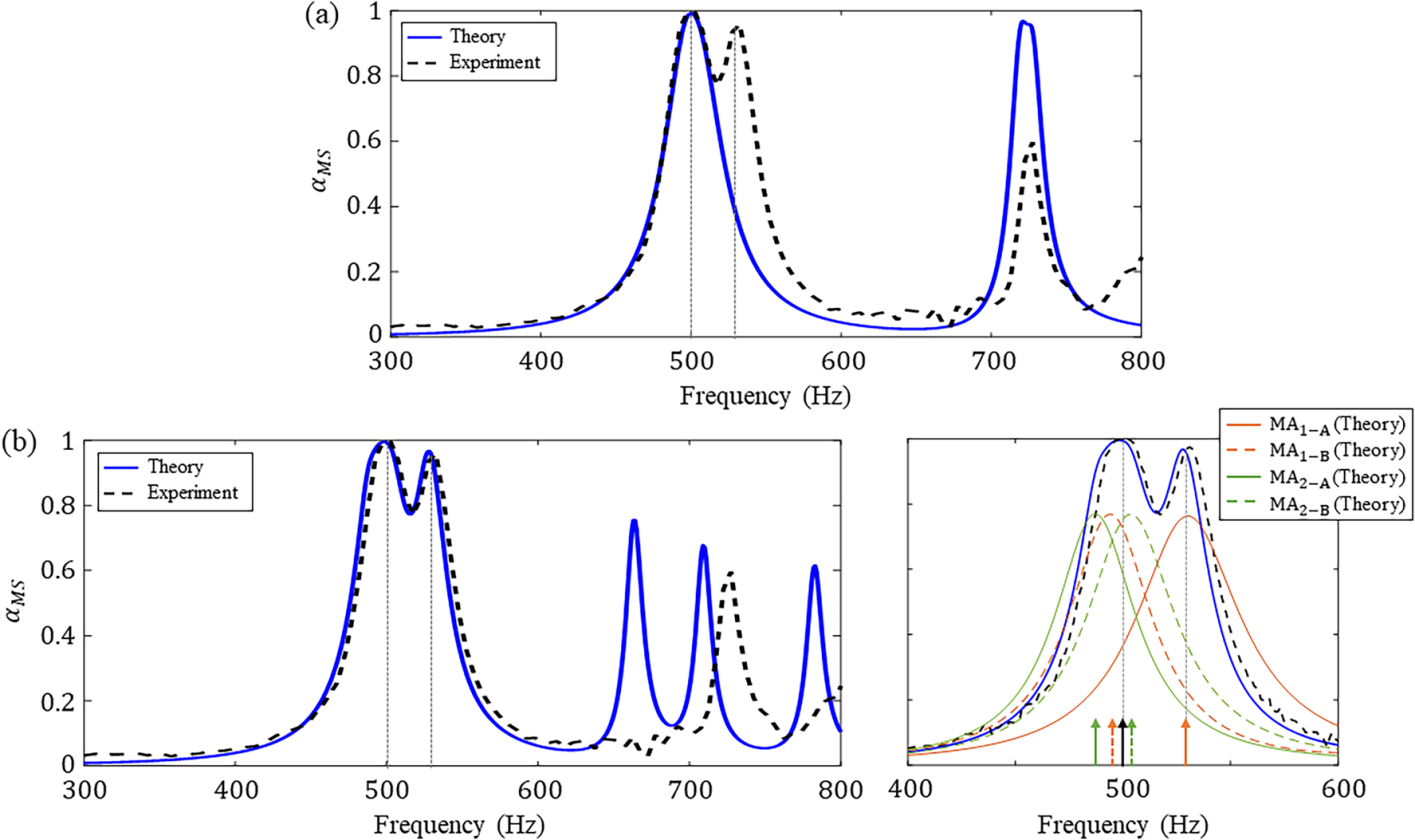
Absorption spectrum (experiment) of the fabricated metasurface. For comparison, the spectra (theory) of (**a**) the designed metasurface and (**b**) the metasurface with the estimated tensions are shown together. The tensions in the membranes of the designed metasurface are (${T}_{1-{\text{A}}}$, ${T}_{1-{\text{B}}}$, ${T}_{2-{\text{A}}}$, ${T}_{2-{\text{B}}}$) = (45, 45, 45, 45) N/m and the estimated tensions are (${T}_{1-{\text{A}}}$, ${T}_{1-{\text{B}}}$, ${T}_{2-{\text{A}}}$, ${T}_{2-{\text{B}}}$) = (66, 39, 44, 53) N/m. The absorption spectra of the MAs with the estimated tensions are indicated as orange (${{\text{MA}}}_{1-{\text{A}}}$ and ${{\text{MA}}}_{1-{\text{B}}}$) and green (${{\text{MA}}}_{2-{\text{A}}}$ and ${{\text{MA}}}_{2-{\text{B}}}$) lines. The arrows correspond to the resonance frequencies of the MAs (orange and green arrows) and the metasurface (black arrow).

Figure 5a compares the experimental and theoretical absorption spectra for the metasurface designed to have (${T}_{1}$, ${T}_{2}$, ${\beta }_{1}$, ${\beta }_{2}$) = (45 N/m, 45 N/m, 0.49, 0.60). The fabricated metasurface with a thickness of λ/17.2 achieved near-perfect absorption of sound, i.e., ${\alpha }_{MS}\ge$ 0.99, at the target frequency of 500 Hz. The experimental bandwidth for 50% sound absorption was 67 Hz. As shown in Fig. 5a, an additional 97% absorption peak for the metasurface was observed at 530 Hz in contrast to the theoretical results. To examine the reason for the occurrence of an additional absorption peak, the tension of the membrane in each MA was estimated from the experimental result. To distinguish between the MAs with the same $\beta$, the four MAs in the unit cell were named by using the subscripts A and B, as follows: ${{\text{MA}}}_{1-{\text{A}}}$, ${{\text{MA}}}_{1-{\text{B}}}$, ${{\text{MA}}}_{2-{\text{A}}}$, and ${{\text{MA}}}_{2-{\text{B}}}$. That is, the membranes in ${{\text{MA}}}_{j-{\text{A}}}$ and ${{\text{MA}}}_{j-{\text{B}}}$ for $j$ = 1, 2 were in the same location but had the different tensions. By substituting the thicknesses of the membranes before and after bolting into the stress–strain equation for the plane stress, the estimated tensions for the metasurface were (${T}_{1-{\text{A}}}$, ${T}_{1-{\text{B}}}$, ${T}_{2-{\text{A}}}$, ${T}_{2-{\text{B}}}$) = (66, 39, 44, 53) N/m. There was a difference in the tensions even between the MAs with the same $\beta$, indicating that it was difficult to fine-tune the tensions to the desired values (${T}_{1-{\text{A}}}$, ${T}_{1-{\text{B}}}$, ${T}_{2-{\text{A}}}$, ${T}_{2-{\text{B}}}$) = (45, 45, 45, 45) N/m. The difference between the estimated and desired values of the tensions caused the additional peak, resulting in the $Q$-factor of 8.1 which was marginally lower than the theoretical result of 11.1.

Figure 5b compares the experimental and theoretical absorption spectra by applying the estimated tensions for the metasurface. The orange and green lines indicate the absorption spectra of the MAs with subscripts 1 and 2, respectively, and the solid and dashed lines are for the MAs with subscripts A and B, respectively. The arrows correspond to the resonance frequencies of the MAs (orange and green arrows) and the metasurface (black arrow). As evident from the resonance frequencies, the first absorption peak at 500 Hz was caused by hybrid resonance, and the second peak at 530 Hz was caused by the local resonance of the ${{\text{MA}}}_{1-{\text{A}}}$. The first and second peaks of the metasurface were induced by the fundamental modes of the MAs. There is a difference between the theoretical and experimental results due to the approximation of the higher-order eigenmodes in the 1D theoretical model. The theoretical and experimental results were in good agreements for the absorption peak induced by the fundamental mode of the MAs, as indicated in Fig. 5b. Therefore, a metasurface can be designed for perfect sound absorption at a desired frequency by using the theoretical model established in this study.

## Discussion

In this paper, we proposed a sound-absorbing metasurface composed of subwavelength HRs with cavities partitioned non-uniformly by membranes. The metasurface was designed with the aid of a theoretical derivation of its frequency-dependent effective acoustic impedance for perfect sound absorption. A theoretical model was formulated including visco-thermal losses in the subwavelength HRs and end corrections due to radiation impedance at the ends of the neck. We enhanced the accuracy of the theoretical model by considering higher-order eigenmodes of the membranes and using mode superposition theory. By optimizing the location of the membrane in each MA and determining delicately the resonance frequency of each MA, the impedance of the metasurface was matched to that of the ambient air so that hybrid resonance between the MAs at a target frequency was induced. As a result, the designed metasurface with a thickness of λ/17.2 absorbed over 99% of the sound energy at the target frequency which is different from the resonance frequencies of the MAs, and the sound absorbing performance was experimentally validated at 500 Hz in an impedance tube. The proposed metasurface yielded a low $Q$-factor with the aid of the hybrid resonance and the visco-thermal losses in the narrow orifices compared to a single resonator, which is advantageous when designing a broadband absorber composed of several different unit cells tuned at different peak frequencies. Near-perfect absorption of sound was achieved by the fabricated metasurface at the target frequency which was the 12.3% lower than that of perfect absorption by previous metasurfaces inducing hybrid resonance between HRs without membranes. This implies that the proposed metasurface can be an alternative solution to reduce noise while occupying less volume in practical applications where sound absorption needs to be improved in limited space. The experimental result was in good agreement with the theoretical one, demonstrating the accuracy of the theoretical model to derive frequency-dependent effective acoustic impedance of the metasurface. So, the theoretical model can be used to design metasurfaces frequency-selectively to achieve perfect absorption of sound. This study also demonstrates the potential of the proposed metasurface for noise mitigation of tonal sound in mechanical systems, as it can absorb sound energy near-perfectly at a specific target frequency. From a more practical point of view, our future works include the followings. Since it is difficult to accurately control the tension, a design approach that is less sensitive to tension is required to enhance performance. For the various mechanical systems that generate noise at multiple frequencies or in a broad frequency range, the metasurface needs to absorb a wider frequency band or multiple frequencies^41–45^. Also, to apply acoustic metasurfaces to outdoor environment with flow, proper theoretical and numerical frameworks need to be studied^46–51^. Expansion from planar surfaces to nonplanar surfaces also would improve the applicability of the proposed metasurface to machines of various shapes^52^.

## Methods

### Simulation setup

For analysis of absorbing mechanism via hybrid resonance, we performed FE simulation with the thermoviscous acoustics module and the solid mechanics module in the commercial software COMSOL Multiphysics 6.0. Acoustically rigid boundary conditions were imposed on the interfaces between air and the structures, except for the membrane. Fixed constraints were set on the edges of the membranes. Silicone rubber was used for the membranes, which has a mass density, Young’s modulus, Poisson’s ratio, and loss factor of ${\rho }_{m}$ = 1100 ${\text{kg}}/{{\text{m}}}^{3}$, ${E}_{m}$ = 1.2 MPa, ${\nu }_{m}$ = 0.48, and ${\eta }_{m}$ = 0.01, respectively.

### Experimental setup

To measure the sound absorption coefficients of the metasurface, we used a circular impedance tube with the diameter of 100 mm (see Fig. 4a). The operating frequency range of the impedance tube spans from 50 to 1700 Hz. A loudspeaker was mounted at the end of the tube to radiate white noise generated by a Brüel and Kjær PULSE Labshop and amplified by a power amplifier. The acoustic pressure signals were measured using two 1/4-inch microphones (GRAS 46BE) in the circular impedance tube. The absorption coefficients were calculated from the pressure data according to the international standard, ISO 10534-2.

## Data Availability

The datasets used and/or analysed during the current study available from the corresponding author on reasonable request.
